# Metallo-β-lactamase domain-containing protein 2 is S-palmitoylated and exhibits acyl-CoA hydrolase activity

**DOI:** 10.1074/jbc.RA120.015701

**Published:** 2020-12-03

**Authors:** Martin Ian P. Malgapo, Jenelle M. Safadi, Maurine E. Linder

**Affiliations:** Department of Molecular Medicine, College of Veterinary Medicine, Cornell University, Ithaca, New York, USA

**Keywords:** MBLAC2, S-palmitoylation, S-fatty acylation, posttranslational modification (PTM), zDHHC enzyme, hydrolase, ACOT, thioesterase, metallo-β-lactamase, ACOT, acyl coenzyme A thioesterase, CoA, Coenzyme A, DDM, dodecylmaltoside, DMEM, Dulbecco’s modified Eagle’s medium, EGFR, epidermal growth factor receptor, MAPK, mitogen-activated protein kinase, MBL, metallo-β-lactamase, ODYA, octadecynoic acid, pacFA, photoactivatable and clickable fatty acid, PEI, Polyethylenimine, PI3K, phosphatidylinositol 3-kinase, RHM, reaction hot mix, Sf9, *Spodoptera frugiperda* cell line, TCEP, tris(2-carboxyethyl) phosphine

## Abstract

Members of the metallo-β-lactamase (MBL) superfamily of enzymes harbor a highly conserved αββα MBL-fold domain and were first described as inactivators of common β-lactam antibiotics. In humans, these enzymes have been shown to exhibit diverse functions, including hydrolase activity toward amides, esters, and thioesters. An uncharacterized member of the human MBL family, MBLAC2, was detected in multiple palmitoylproteomes, identified as a zDHHC20 S-acyltransferase interactor, and annotated as a potential thioesterase. In this study, we confirmed that MBLAC2 is palmitoylated and identified the likely S-palmitoylation site as Cys254. S-palmitoylation of MBLAC2 is increased in cells when expressed with zDHHC20, and MBLAC2 is a substrate for purified zDHHC20 *in vitro*. To determine its biochemical function, we tested the ability of MBLAC2 to hydrolyze a variety of small molecules and acylprotein substrates. MBLAC2 has acyl-CoA thioesterase activity with kinetic parameters and acyl-CoA selectivity comparable with acyl-CoA thioesterase 1 (ACOT1). Two predicted zinc-binding residues, Asp87 and His88, are required for MBLAC2 hydrolase activity. Consistent with a role in fatty acid metabolism in cells, MBLAC2 was cross-linked to a photoactivatable fatty acid in a manner that was independent of its S-fatty acylation at Cys254. Our study adds to previous investigations demonstrating the versatility of the MBL-fold domain in supporting a variety of enzymatic reactions.

Protein S-palmitoylation refers to the reversible posttranslational addition of palmitate to cysteine residues of a protein *via* a thioester bond. In general, the palmitate moiety serves as a membrane anchor for proteins that lack transmembrane domains. Functionally, S-palmitoylation regulates the trafficking, stability, and activity of many peripheral and integral membrane proteins (1, 2, 3). To date, over 10,000 palmitoylated proteins have been catalogued in the SwissPalm database (4). It is estimated that over 1200 human genes (10% of the human genome) encode for palmitoylated proteins (5). A substantial fraction of these proteins has been functionally characterized and implicated in the pathogenesis of a variety of human diseases, including disorders in the nervous system, cancer, and aberrant cellular metabolic processes (5, 6, 7, 8). However, the biological relevance of many other palmitoylated proteins has yet to be uncovered, as is the case of the uncharacterized protein metallo-β-lactamase domain-containing protein 2 (MBLAC2).

Evidence for the S-palmitoylation of the human ortholog of MBLAC2 exists in 8 of 17 published proteomic studies utilizing various biochemical techniques in several cell types (*e.g.,* platelets, B-cells, HEK-293 cells) (4). However, MBLAC2 S-palmitoylation has not been validated, nor its S-palmitoylation site/s mapped. Furthermore, the S-palmitoyl transferases that catalyze MBLAC2 S-palmitoylation have not been identified. Recently, a comprehensive human interactome screen using affinity purification–mass spectrometry reported an interaction between MBLAC2 (bait) and the S-palmitoyl transferase zDHHC20 (hit) in HEK-293 cells, suggesting that MBLAC2 is a potential substrate of zDHHC20 (9).

Structurally informed sequence analysis reveals that MBLAC2 belongs to the metallo-β-lactamase (MBL) superfamily of enzymes characterized by a highly conserved metal-bound αββα fold. This MBL fold was first observed in prokaryotic enzymes that initiate the hydrolysis and inactivation of common β-lactam antibiotics but is now recognized to be widespread in biology. Of the 34,000 MBL-fold enzymes, only about 1000 retain the classical antibiotic resistance activity as β-lactamases. The remaining enzymes have been implicated in various biological processes such as cell detoxification pathways, metabolism, and nucleic acid modifications (10), highlighting the prevalence and versatility of the MBL-fold domain in supporting a variety of enzymatic reactions.

In humans, eighteen MBL-fold proteins exhibit diverse sequences with as little as 25% identity between some enzymes (11). Phylogenetic analyses cluster these proteins into three groups. Group 1 includes MBLAC2 and six other proteins. To date, four Group 1 MBL-fold proteins have been assigned a biochemical function: hydroxyacylglutathione hydrolase, commonly called glyoxalase II, catalyzes a key step in the detoxification of 2-oxoaldehydes (12, 13), ethylmalonic encephalopathy protein 1 (ETHE1) metabolizes the toxic H_2_S gas in the mitochondria (14), β-lactamase-like protein 2 (LACTB2) acts as a mitochondrial endonuclease (15), and metallo-β-lactamase domain-containing protein 1 (MBLAC1) serves as a specific, high-affinity target for the glutamate transporter inducer, ceftriaxone (16), and has recently been characterized as an endonuclease that is selective for 3’ processing of replication-dependent histone pre-mRNA (17). The biochemical functions of MBLAC2, hydroxyacylglutathione hydrolase-like (HAGHL), and paroxysmal nonkinesigenic dyskinesia (PNKD) proteins are still unknown. The remaining members of the human MBL family also display diverse biological functions. For example, the DNA cross-link repair 1 protein (DCR1 A, B) and cleavage-polyadenylation specificity factor 73 (CPSF73) proteins are involved in DNA repair pathways and RNA processing (10, 18, 19). These enzymes contain an additional β-CPSF-Artemis-SNM1-Pso2 (β-CASP) domain, which along with the MBL-fold domain is important in nucleic acid binding and nuclease catalysis (18, 20).

In this study, we confirmed MBLAC2 S-palmitoylation and its interaction with zDHHC20. We investigated the enzymatic function of MBLAC2 by testing it for hydrolase activity toward amide- and thioester-linked substrates, seeking insight into how its status as a palmitoylated protein might be linked to its enzymatic function.

## Results

### MBLAC2 S-palmitoylation is dependent on Cys254

**Figure 1:**
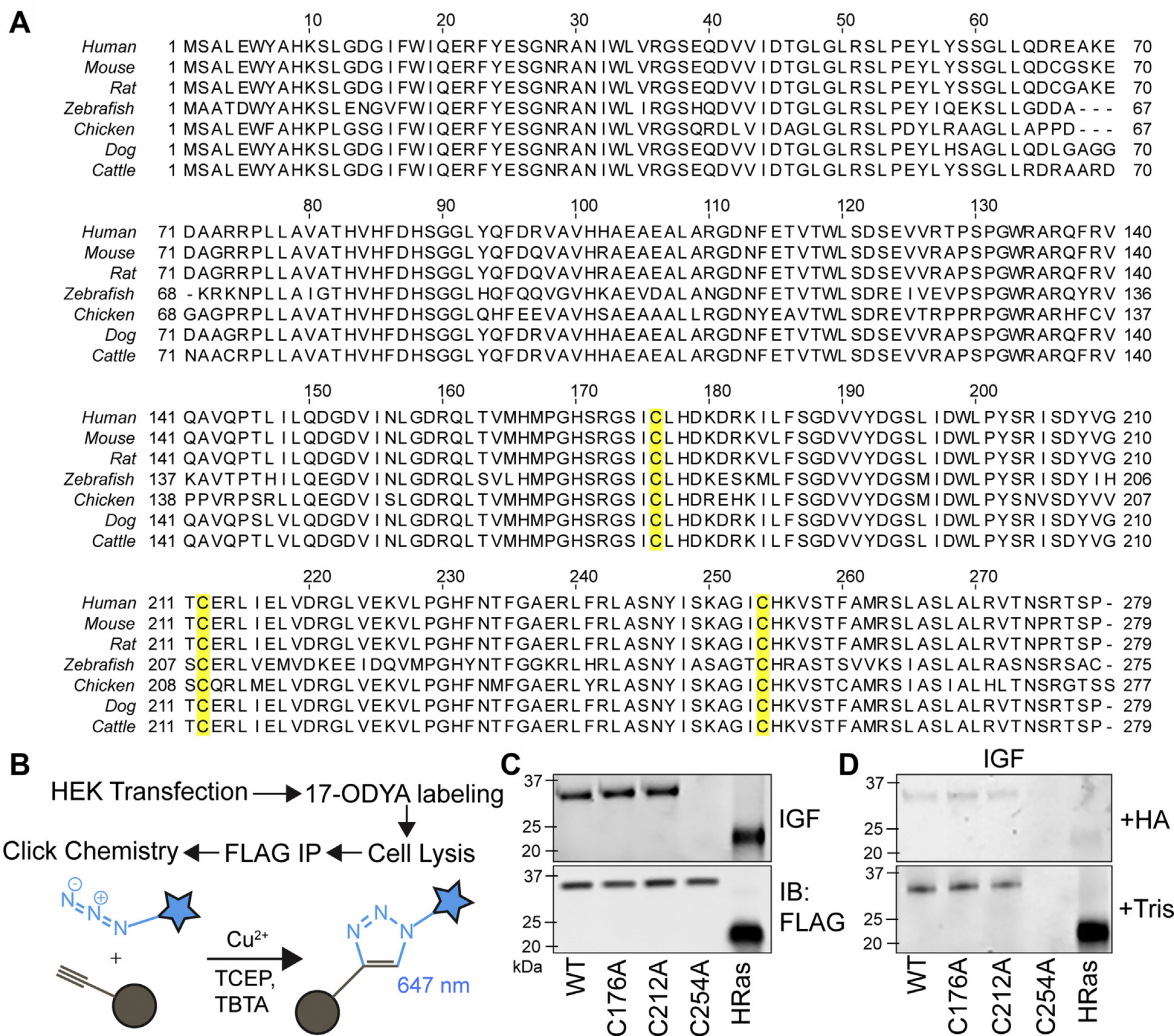
**MBLAC2 is S-palmitoylated at C254.***A*, sequence alignment reveals three highly conserved cysteines in vertebrate orthologs of MBLAC2: Cys176, Cys212, and Cys254 (*highlighted in yellow*). *B*, schematic diagram of the click chemistry method used to detect the S-palmitoylation of FLAG-MBLAC2 in HEK-293 cells. In this technique, palmitate is exchanged with an alkyne-containing analog, 17-ODYA. The alkyne functional group then undergoes a cycloaddition reaction with a fluorescent azide. *C*, S-palmitoylation levels of FLAG-MBLAC2 are measured by in-gel fluorescence (IGF) at 647 nm. Protein levels are compared by immunoblotting (IB) using a FLAG antibody. HRas, a dually palmitoylated protein, was used as a positive control. The gel is representative of at least three independent experiments using both FLAG-tagged and GFP-tagged MBLAC2 constructs. *D*, ODYA labeling of FLAG-MBLAC2 is sensitive to hydroxylamine. In-gel fluorescence of ODYA-labeled proteins was detected after treatment with 1 M hydroxylamine (HA) or 1 M Tris. The gel is representative of at least three independent experiments.

The SwissPalm S-palmitoylation database annotates MBLAC2 as a palmitoylated protein, based on its detection in multiple palmitoyl-proteome studies (4). Sequence alignment of human MBLAC2 and its vertebrate orthologs shows three highly conserved cysteines in its amino acid sequence that could potentially undergo S-palmitoylation: Cys176, Cys212, and Cys254 (Fig. 1*A*). To identify which of these cysteines is/are palmitoylated, we created N-terminally FLAG-tagged constructs of the wildtype, along with individual cysteine mutants and expressed them in HEK-293 cells. Using click chemistry, we measured incorporation of 17-octadecynoic acid (ODYA), a palmitate analog, into each of these constructs (Fig. 1*B*). The significant reduction in the 17-ODYA labeling of the C254A mutant demonstrated that S-palmitoylation is dependent upon Cys254, suggesting that it is the main site of S-palmitoylation. Mutation of either Cys176 or Cys212 into an alanine residue did not affect MBLAC2 S-palmitoylation (Fig. 1*C*). A similar loss of S-palmitoylation of the Cys254 mutant was observed with N-terminally FLAG-tagged constructs using an acyl biotin exchange assay (M.I.P.M. and M.E.L., unpublished results). Incorporation of 17-ODYA into MBLAC2 was sensitive to hydroxylamine treatment, consistent with a thioester linkage of the fatty acid analog (Fig. 1*D*).

### zDHHC20 increases the S-palmitoylation of MBLAC2 in HEK-293 cells and *in vitro*

**Figure 2:**
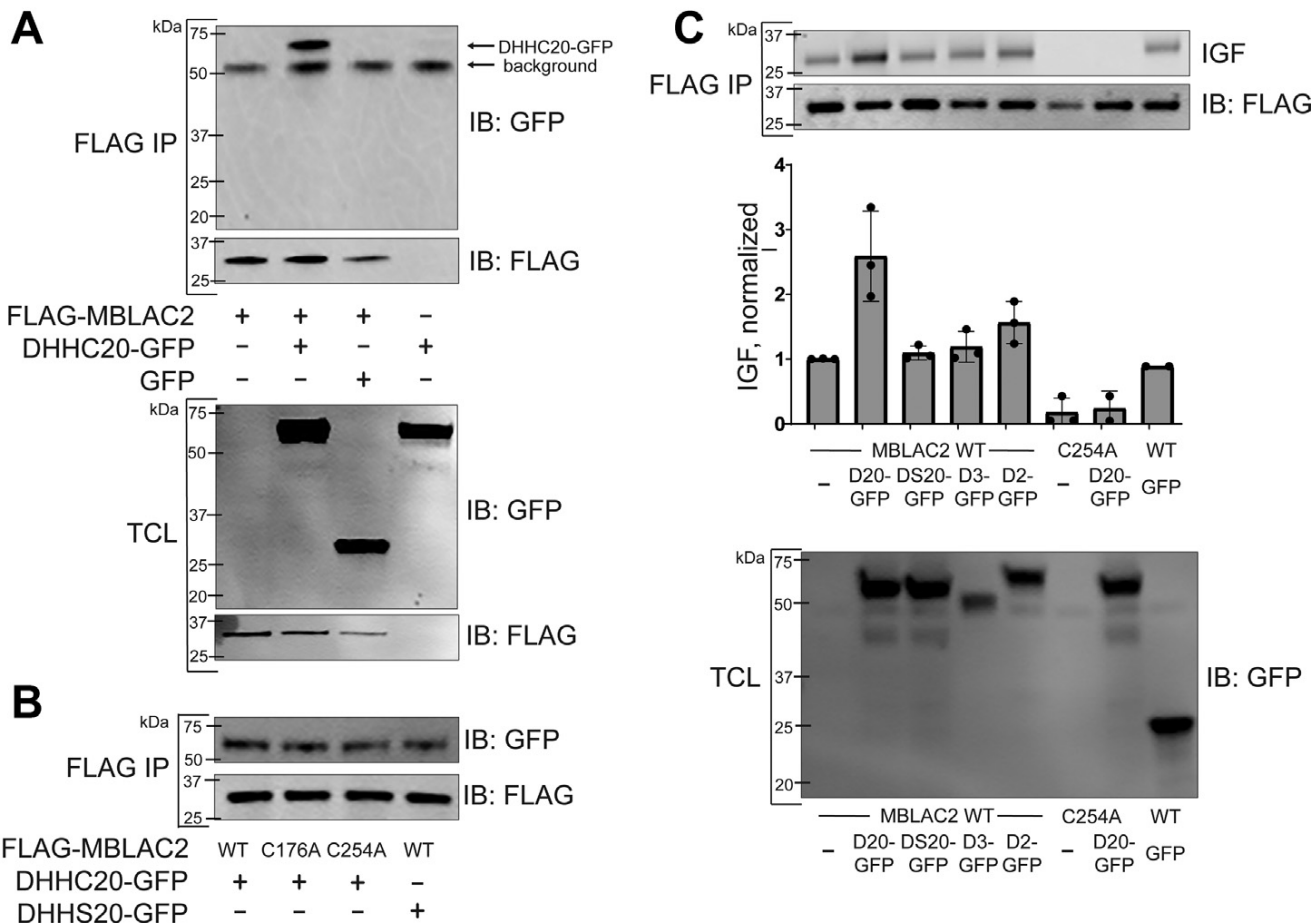
**zDHHC20 interacts with and increases the S-palmitoylation of MBLAC2 when coexpressed in HEK-293 cells.***A*, FLAG-MBLAC2 and zDHHC20-GFP were coexpressed in HEK-293 cells. After cell lysis, FLAG-MBLAC2 was purified using FLAG beads. The FLAG immunoprecipitates (IPs) were resolved by SDS-PAGE and visualized in immunoblots with FLAG antibody to detect MBLAC2 or GFP antibody to detect zDHHC20. The protein levels in the total cell lysates (TCLs) were also compared by immunoblotting with the appropriate antibodies. The figure is representative of two independent experiments. *B*, MBLAC2 association with zDHHC20 does not require the S-palmitoylation site in MBLAC2 (C254) or catalytic activity of zDHHC20 (zDHHS20). FLAG immunoprecipitates were analyzed as decribed for *A*, The figure is representative of two independent experiments. *C*, S-palmitoylation of MBLAC2 was assayed as in the legend to Figure 1 following coexpression of wildtype MBLAC2 or S-palmitoylation-deficient mutant MBLAC2(C254A) with zDHHC20-GFP (D20), catalytically inactive zDHHS20-GFP (DS20), zDHHC3-GFP (D3), and zDHHC2-GFP (D2) and detected by in-gel fluorescence. zDHHC-GFP protein expression in cell lysates was detected by anti-GFP immunoblots. The data shown are representative of three independent experiments. Data are displayed as mean ± S.E.M., n = 3.

We then investigated if the observed S-palmitoylation in MBLAC2 was catalyzed by the zDHHC enzymes, a family of palmitoyl transferases responsible for S-linked fatty acylation of protein substrates. An interactome screen in HEK-293 cells reported that MBLAC2 interacts with zDHHC20 (9). We validated this interaction by showing that N-terminally FLAG-tagged MBLAC2 pulled down C-terminally GFP-tagged zDHHC20 when the two proteins were coexpressed in HEK-293 cells (Fig. 2*A*). Of interest, this interaction did not require the palmitoylated cysteine (C254) in MBLAC2 or the enzymatic activity of zDHHC20, in which the catalytic cysteine of zDHHC20 was mutated to serine (zDHHS20) (Fig. 2*B*). Together, these results suggest that the S-palmitoylation process is not crucial for the interaction of MBLAC2 with zDHHC20. Notably, coexpression of FLAG-MBLAC2 with zDHHC20-GFP, but not with zDHHS20-GFP, in HEK-293 cells resulted in roughly a threefold increase in the S-palmitoylation level of wildtype MBLAC2 (Fig. 2*C*). As expected, the S-palmitoylation level of MBLAC2(C254A) remained at background level and was not affected by coexpression with zDHHC20-GFP. Of interest, coexpression of FLAG-MBLAC2 with either zDHHC2-GFP or zDHHC3-GFP resulted in a minimal or no increase in MBLAC2 S-palmitoylation.

**Figure 3:**
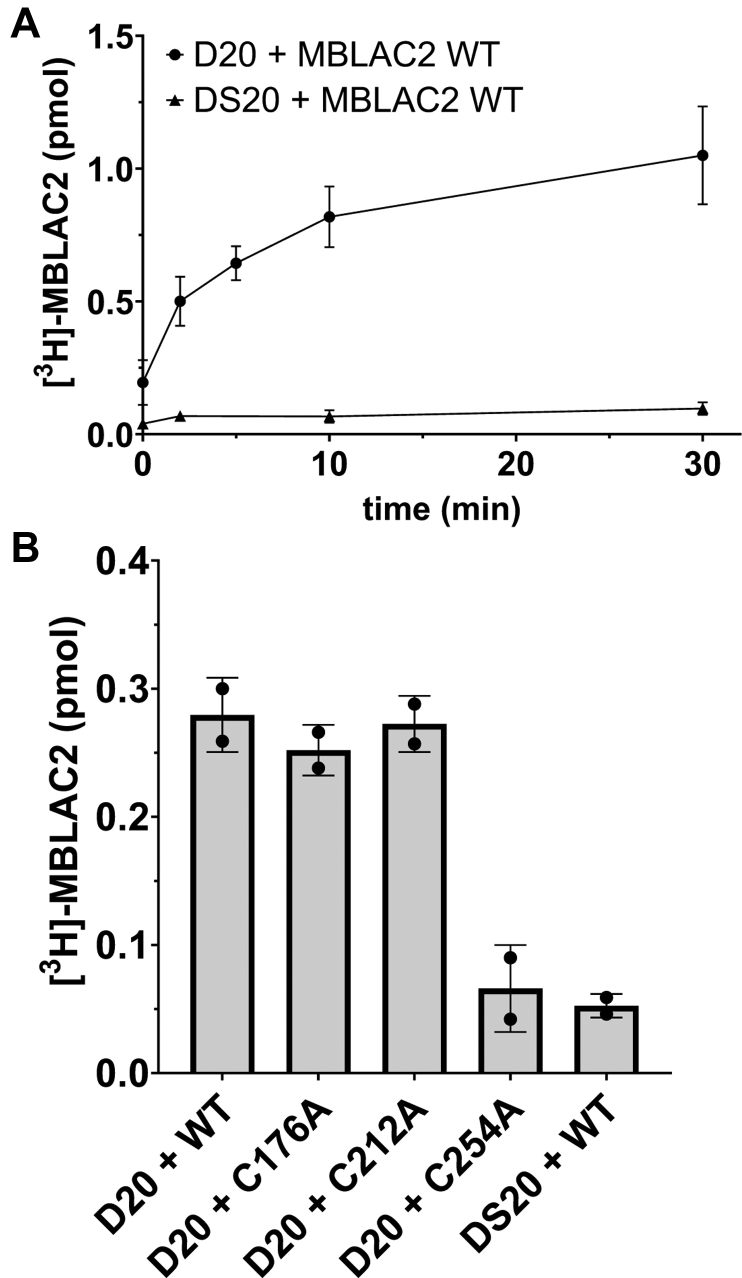
**zDHHC20 S-palmitoylates wildtype MBLAC2 *in vitro*.***A*, zDHHC20 (50 nM) or catalytically inactive zDHHS20 (50 nM) was incubated with MBLAC2 (1 μM) and [^3^H]-palmitoyl CoA (1 μM) at 25 °C for the indicated times. The reaction was stopped, and [^3^H]-palmitate incorporation was quantified by scintillation spectroscopy as described under Experimental Procedures. DS20, catalytically inactive zDHHC20. Data are displayed as the mean ± S.E.M., n = 2. *B*, zDHHC20 was incubated with wildtype MBLAC2 or MBLAC2 with cysteine point mutations at 25 °C for 10 min, then processed as described for *A*, Data are displayed as the mean ± S.E.M., n = 2.

Using purified enzyme preparations, we found that zDHHC20, but not zDHHS20, palmitoylated wildtype MBLAC2 *in vitro*, confirming that MBLAC2 is a zDHHC20 substrate (Fig. 3*A*). Mutation of Cys254, but not Cys176 or Cys212, significantly diminished zDHHC20-mediated S-palmitoylation of the MBLAC2 protein (Fig. 3*B*), providing further support that Cys254 is the relevant S-palmitoylation site. Our *in vitro* results are consistent with MBLAC2 being a substrate of zDHHC20, but it cannot be ruled out that other zDHHC enzymes might have palmitoyl transferase activity for MBLAC2.

### S-palmitoylation does not affect the endomembrane distribution of MBLAC2

**Figure 4:**
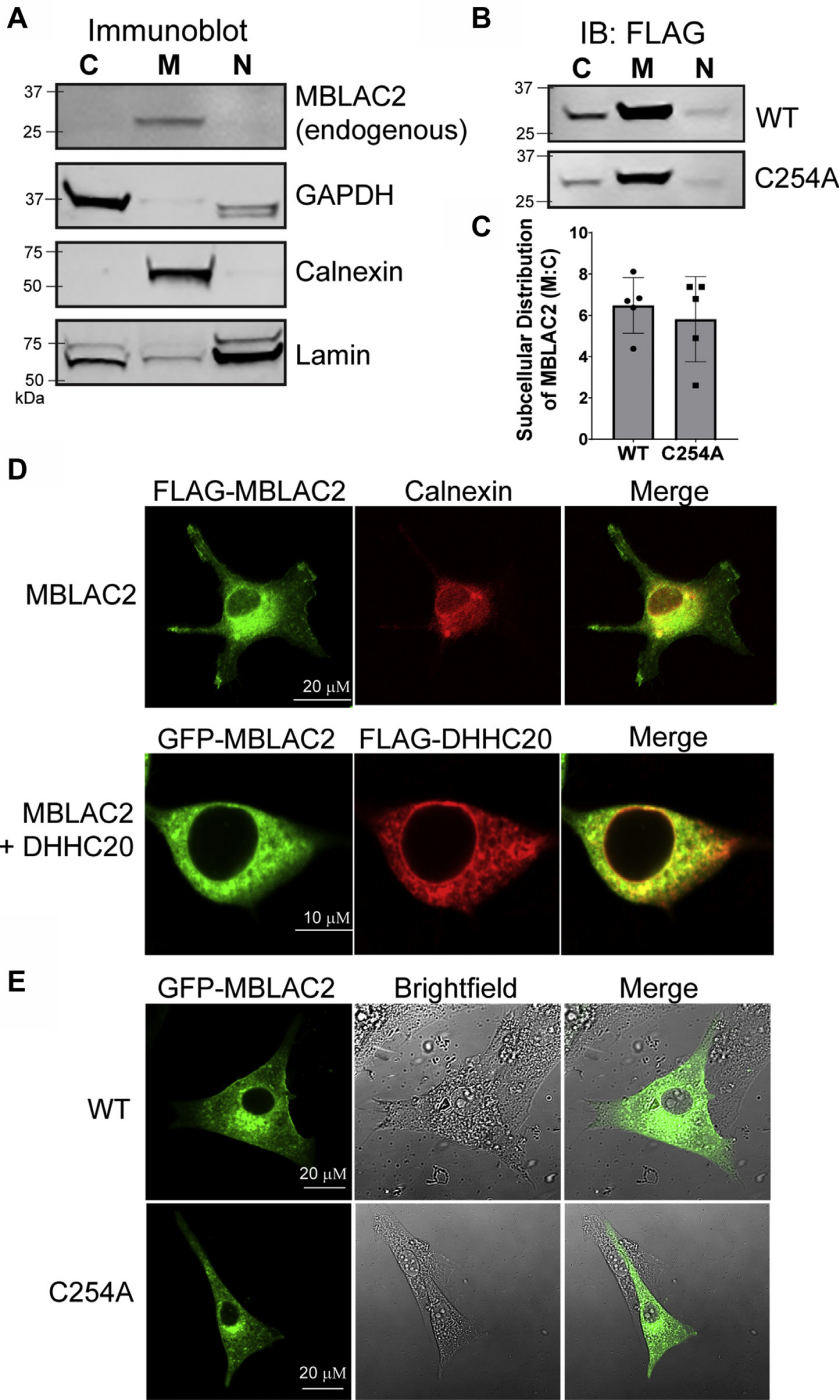
**S-palmitoylation does not impact endomembrane localization of MBLAC2.***A*, HEK-293 cells were subjected to subcellular fractionation (16). The cytosol (C) was released by digitonin permeabilization of the plasma membrane and retained separately; membranes (M) were then solubilized with NP-40 and the detergent extract was collected. Finally, the detergent-insoluble fraction was lysed with SDS and designated as nuclear (N) fraction. Endogenous MBLAC2, calnexin (ER), GAPDH (cytosol), and lamin (nuclear) were detected in immunoblots. The data shown are representative of three independent experiments. *B*, HEK-293 cells were transfected with either wildtype FLAG-MBLAC2 or S-palmitoylation-deficient mutant MBLAC2 (C254A) and fractionated as described for *A*. MBLAC2 was detected by immunoblotting with the FLAG antibody. The experiment shown is representative of at least four independent experiments. *C*, quantification of the subcellular distribution of wildtype and S-palmitoylation-deficient MBLAC2. Data are displayed as the mean ± SEM, n = 5. *D*, when expressed in U87 cells, FLAG-MBLAC2 (*green*) immunofluorescence codistributes with that of calnexin (ER, *red*). Image is representative of six cells from two independent experiments. GFP-MBLAC2 (*green*) colocalizes with FLAG-zDHHC20 (*red*) when cotransfected in HEK-293 cells. Image is representative of at least 20 cells from three independent experiments. *E*, GFP-MBLAC2 WT and GFP-MBLAC2(C254A) display similar localization patterns. GFP-epifluorescence was detected on fixed, unpermeabilized U87 cells. Images are representative of at least six cells from two independent experiments.

The subcellular localization of MBLAC2 has not been examined. To address this, we first performed subcellular fractionation of HEK-293 cells to assess the distribution of endogenous MBLAC2. MBLAC2 cofractionated with the ER protein calnexin, consistent with its enrichment in cell membranes (Fig. 4*A*). Under conditions of overexpression, MBLAC2 was primarily associated with the membrane fraction, with some protein in both the cytoplasm and the nuclear fraction (Fig. 4*B*). As S-palmitoylation facilitates a controlled association of a soluble protein with a lipid bilayer, we tested whether S-palmitoylation mediated MBLAC2’s membrane association. Surprisingly, the S-palmitoylation-deficient mutant, MBLAC2(C254A) was similarly enriched in the membrane fraction as the wildtype protein (Fig. 4, *B*–*C*), suggesting that loss of S-palmitoylation does not result in a pronounced redistribution of MBLAC2 to the cytoplasm or the nucleus. These results suggest that a different structural feature of MBLAC2 mediates its membrane attachment.

To identify the membrane compartment where MBLAC2 resides, we expressed FLAG-MBLAC2 or GFP-MBLAC2 in U87 and HEK-293 cells and visualized its localization by immunofluorescence imaging or epifluorescence on fixed cells (Fig. 4, *D*–*E*). MBLAC2 displayed a reticular staining that extended throughout the cell and that overlapped extensively with that of the ER marker calnexin. Some plasma membrane staining was also evident. Consistent with MBLAC2-zDHHC20 interactions ((9), Fig. 2), MBLAC2 immunofluorescence also overlapped with that of zDHHC20. The S-palmitoylation-deficient mutant of MBLAC2 was localized similarly to the wildtype protein as seen by GFP epifluorescence in fixed cells (Fig. 4*E*) or when FLAG-tagged or untagged constructs were visualized by immunofluorescence with FLAG or MBLAC2 antibodies, respectively (M.I.P.M. and M.E.L., unpublished results). The imaging data further confirms that the localization of MBLAC2 is independent of its S-palmitoylation.

### MBLAC2 exhibits acyl-CoA thioesterase activity similar to type I ACOTs

MBLAC2 phylogenetically belongs to the human MBL-fold family of enzymes. Several members of this enzyme family have been characterized and shown to exhibit hydrolase activity toward a diverse set of functional groups such as amides, esters, and thioesters. To study the activity of MBLAC2 *in vitro*, we made purified protein preparations of the MBLAC2 enzyme. Recombinant MBL proteins are typically purified from bacterial cell cultures. However, MBLAC2 protein produced in bacteria was insoluble. Accordingly, we engineered a recombinant baculovirus of MBLAC2 that included a hexahistidine tag in the N terminus and a FLAG epitope in the C terminus to aid in protein purification. This eukaryotic expression system also had the advantage of maintaining S-palmitoylation of MBLAC2. MBLAC2 expressed in *Spodoptera frugiperda* (Sf9) insect cells and extracted using the nonionic detergent dodecylmaltoside (DDM) was highly purified and enzymatically active after successive Ni-affinity and FLAG-affinity purification.

We initially tested the ability of MBLAC2 to hydrolyze two small molecules already known to be substrates of some related MBL-fold proteins: nitrocefin, a cephalosporin β-lactam antibiotic substrate routinely used to detect β-lactamase activity in microbes, and S-D-lactoyl glutathione, the physiological intermediate in the metabolism of various toxic aldehydes by the hydroxyacylglutathione hydrolase enzyme. These substrates contain an amide and a thioester functional group, respectively. In addition, because of the reported association of human MBLAC1 with glutamate transport (16), we tested if MBLAC2 could hydrolyze the amide-containing small molecule glutamine. Our purified preparations of MBLAC2 showed little to no hydrolase activity toward all the substrates tested (Fig. S1). Recently, Diene *et al.* reported that MBLAC2 has β-lactamase activity toward nitrocefin, a common β-lactam antibiotic (21). However, the kinetic values reported (*k*_cat_, 0.0004 s^-1^; *K*_*m*_, 370 μM) were significantly lower compared with other established β-lactamases. Although we could detect nitrocefin hydrolase activity in a partially purified preparation of MBLAC2 (Ni pool), this activity was lost upon further purification by FLAG affinity chromatography (Fig. S1*A*). Thus, we are not able to confirm that MBLAC2 has β-lactamase activity.

S-palmitoylation is a unique feature of MBLAC2 among the human MBL-fold proteins. None of the other 17 human MBL-fold proteins are annotated in the SwissPalm database as S-palmitoylated proteins (4). Of interest, known protein depalmitoylases, acyl protein thioesterase 1 (APT1) and the α/β hydrolase domain protein 17 (ABHD17), are themselves palmitoylated proteins. Because of the association of MBLAC2 with S-palmitoylation, we sought to determine whether MBLAC2 has protein depalmitoylase activity. Two palmitoylated protein substrates: the small G protein HRas that contains two palmitoylated cysteines and the N-myristoylated SH4-GFP fusion protein (22), a model S-palmitoylation substrate that contains three cysteine residues. MBLAC2 did not show protein thioesterase activity toward SH4-GFP radiolabeled with [^3^H]-palmitate (Fig. S1*D*). In addition, MBLAC2 was only able to remove palmitate from ODYA-labeled HRas at high enzyme concentration (∼1 μM), suggesting that the reaction was not catalytic (Fig. S1*E*).

**Figure 5:**
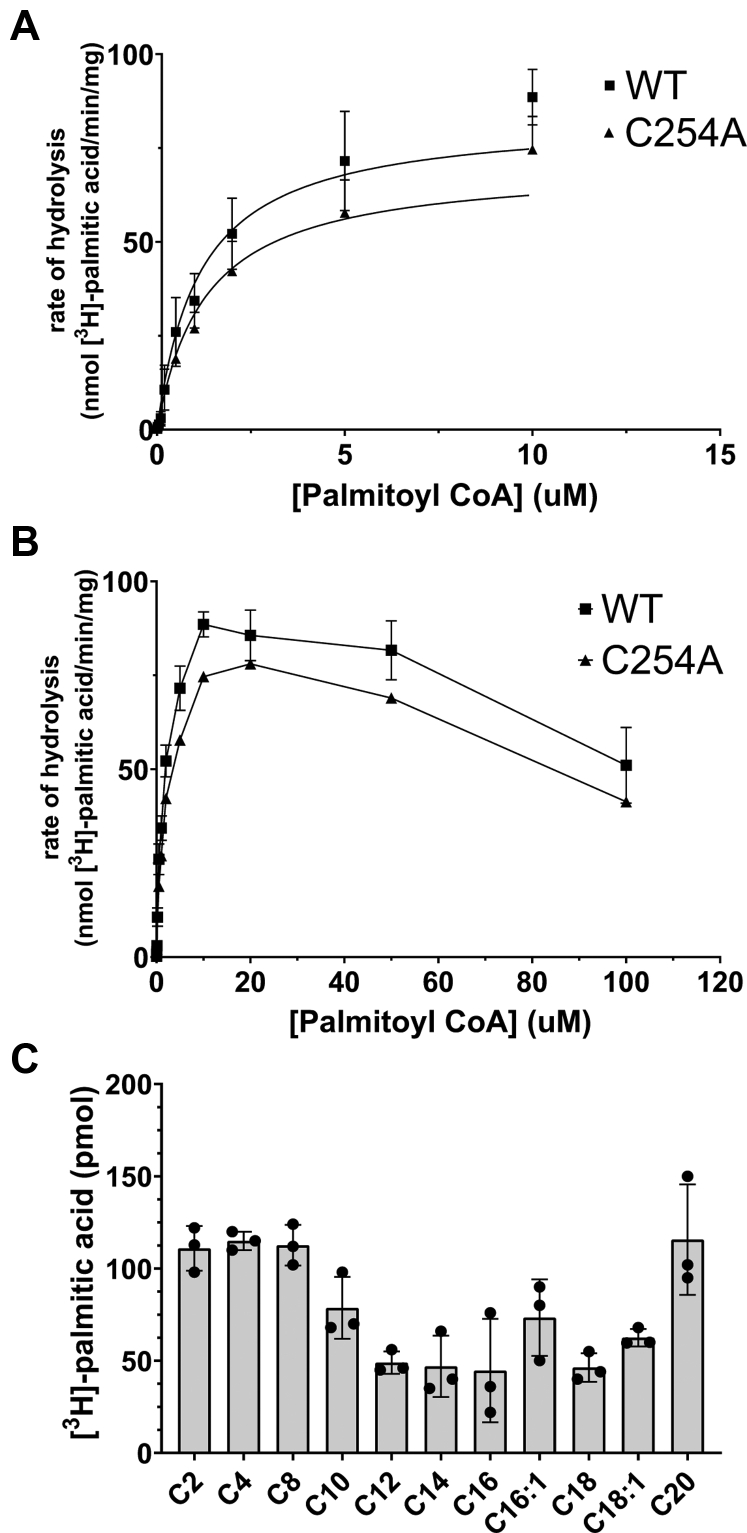
**Palmitoyl CoA hydrolase activity of wildtype and MBLAC2(C254A).***A*, purified wildtype or MBLAC2(C254A) at the indicated concentrations was incubated with [^3^H]-palmitoyl CoA (specific activity 500 dpm/pmol) for 10 min. The reaction was quenched and the [^3^H]-palmitate product was extracted and quantified as described in Experimental Procedures. The proteins exhibit Michaelis–Menten kinetics between 0 to 10 μM palmitoyl CoA concentration. Data are expressed as the mean ± S.E.M., n = 4. *B*, wildtype and MBLAC2(C254A**)** proteins are inhibited by high concentrations of palmitoyl CoA. Same experiments as described for A, but data shown include higher palmitoyl CoA concentrations. Data are expressed as the mean ± S.E.M., n = 4. *C*, acyl chain length selectivity of MBLAC2 (end point assay). MBLAC2 (50 nM) was incubated in the presence of [^3^H]-palmitoyl CoA (1 μM) and unlabeled acyl CoAs for 10 min at 30 °C. The [^3^H]-palmitic acid produced by the hydrolysis reaction was then extracted and quantified as described in Experimental Procedures. Data represent the mean ± S.E.M., n = 3.

The lack of a conclusive hydrolase activity of MBLAC2 toward palmitoylated protein substrates prompted us to look at small molecules that contain thioester-linked palmitate as potential substrates. zDHHC palmitoyl transferases use palmitoyl coenzyme A (CoA) as a fatty acid donor both *in vitro* and in cells (23, 24). MBLAC2 displayed robust thioesterase activity toward palmitoyl CoA (Fig. 5*A*). S-palmitoylation-deficient MBLAC2 had similar activity, suggesting that S-palmitoylation does not have a major effect on the ability of MBLAC2 to act as an acyl-CoA hydrolase *in vitro* (Fig. 5, *A* and *B*), with the caveat that the stoichiometry of S-palmitoylation of our purified MBLAC2 is unknown. The measured kinetic parameters showed only a slight reduction in the *V*_max_ and a small increase in the *K*_*m*_ for the C254A mutant compared with wildtype MBLAC2 (Table 1).

**Table 1:** Kinetic parameters of wildtype MBLAC2 and MBLAC2 (C254A) as palmitoyl-CoA hydrolase (n = 4) compared with ACOT1

Kinetic parameter	WT	C254A	ACOT1 (28)
*K*_*m*_ (μM)	1.9 ± 0.6	2.2 ± 0.7	3.6 ± 2.6
*V*_max_ (nmol/min/mg)	102.5 ± 10.1	87.9 ± 9.5	691 ± 42

To date, thioesterase activity toward palmitoyl CoA and other fatty acyl CoAs has been largely attributed to a family of enzymes called acyl coenzyme A thioesterases (ACOTs) (25, 26, 27). In contrast to generic thioester hydrolases that cleave the thioester bond between a sulfur atom and carbonyl, most ACOTs specifically act on molecular substrates that contain CoA. There are two general types of ACOTs characterized by their response to peroxisome proliferator treatment and the fold of the catalytic active site. The kinetic parameters we measured for the palmitoyl CoA hydrolase activity of MBLAC2 are similar to those obtained for type I ACOTs. Members of type I ACOT enzymes are thioesterases that contain an α/β-hydrolase domain in the active site. The pioneering member and best characterized enzyme in this family is ACOT1, a cytosolic enzyme. The published kinetic parameters for ACOT1 for palmitoyl CoA are comparable with the kinetic parameters we measured for MBLAC2 (Table 1) (28). Both wildtype and MBLAC2(C254A) proteins were inhibited by high concentrations of palmitoyl CoA, similar to some type 1 ACOTs (28, 29). Because of this substrate inhibition, the kinetic parameters for MBLAC2 were calculated using palmitoyl CoA concentrations between 0 and 10 μM.

ACOT1 exhibits substrate selectivity for long-chain (C12-C20) saturated and monounsaturated acyl CoAs (28). We examined the substrate selectivity of MBLAC2 using a competition assay in which a 10-fold excess of nonradiolabeled acyl CoAs of various chain lengths and saturations was added to the reaction with [^3^H]-palmitoyl CoA. MBLAC2 displayed a preference for C12 to C18 fatty acids, with only modest differences observed between unsaturated and monounsaturated fatty acids (Fig. 5*C*). Of interest, this acyl chain length selectivity we measured for MBLAC2 highly parallels that observed for zDHHC20 (30) and is similar to that reported for ACOT proteins (28).

### Aspartate and histidine residues predicted to bind zinc in MBLAC2 are essential for enzyme activity

**Figure 6:**
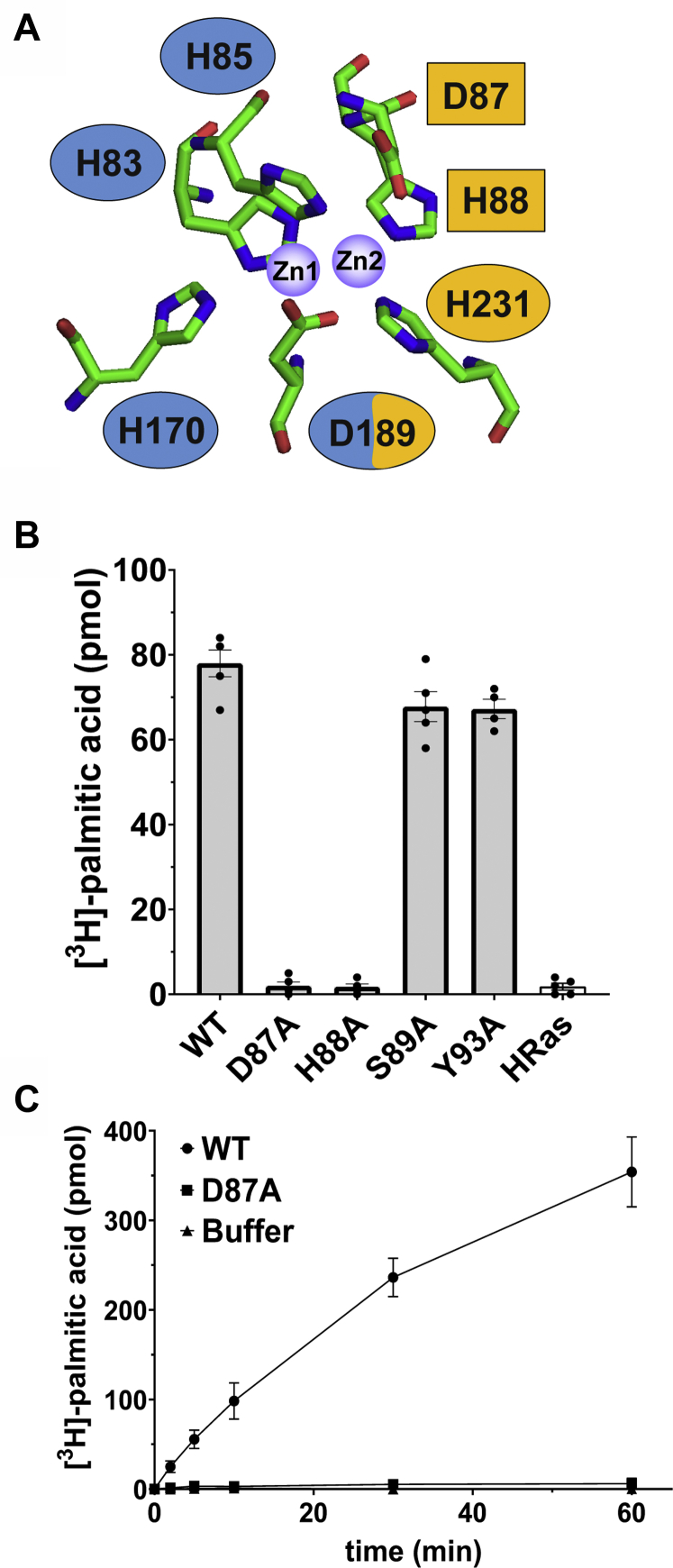
**Residues predicted to bind zinc are required for the acyl-CoA hydrolase activity of MBLAC2.***A*, schematic of the predicted zinc-binding sites of MBLAC2. Zn1-binding residues are in *blue* (H83, H85, H170, D189); Zn2-binding residues are in *yellow* (D87, H88, D189, H231). Single point mutations to alanine were generated. Mutations that resulted in proteins that expressed well in HEK-293 cells and could be solubilized with nonionic detergent are shown as *rectangles*. Mutations that resulted in low protein expression or yielded an insoluble protein are shown as *ovals*. *B*, acyl-CoA hydrolase activity of wildtype and MBLAC2 mutants immunoprecipitated using FLAG beads from transfected HEK-293 cells. MBLAC2 D87A and H88A are single point mutations of Zn2-binding residues; S89A and Y93A were used as controls to assess whether any mutation in proximity to the predicted Zn2-binding site would inactivate acyl-CoA hydrolase activity. HRas was used as a negative control. Data represent the mean ± S.E.M., n = 5. *C*, Time course of acyl-CoA hydrolase activity of wildtype MBLAC2 and D87A mutant purified to near homogeneity from insect cells. Data represent the mean ± S.E.M., n = 4.

We hypothesized that the mechanism of acyl-CoA hydrolysis by MBLAC2 would involve amino acid residues analogous to those observed in the active sites of other MBL enzymes that bind zinc. Bacterial metallo-β-lactamases inactivate β-lactam drugs through a noncovalent mechanism in which one or two equivalents of zinc ions in the active site polarize a water molecule to form a nucleophilic hydroxide ion. The activated nucleophile then attacks the electrophilic carbon of the β-lactam ring transforming it into a ring-open product that is no longer active as an antibiotic. Variations in the chemical identity of the zinc coordination shell become the basis of classifying these bacterial MBL enzymes into three subclasses. Although the structure of MBLAC2 has not been solved, the enzyme is predicted to belong to subclass B3 of MBL enzymes and harbor two zinc ions (11). Zn1 is predicted to bind His83, His85, His170, and Asp189, and Zn2 is predicted to bind Asp87, His88, Asp189, and His231 (Fig. 6*A*).

We examined the significance of these predicted zinc-binding residues in the catalytic activity of MBLAC2 by generating alanine substitutions of each of these residues and expressing the mutant proteins in HEK-293 cells. Mutations of five of these residues individually resulted in low expression and insoluble proteins that could not be further purified and assayed (H83, H85, H170, D189, and H231). Mutations D87A and H88A, both of which are predicted Zn2-binding residues, yielded a detergent-extractable protein that was amenable to FLAG affinity purification from HEK-293 cells. Each of these mutations resulted in a protein in which palmitoyl-CoA hydrolase activity was decreased to near background levels (Fig. 6*B*). These data strongly suggest that the Zn2-binding pocket is crucial for the acyl-CoA hydrolase activity of MBLAC2. To exclude the possibility that any point mutation caused a gross structural perturbation in the protein, we also purified proteins with mutations in amino acids near the Zn2-binding pocket. Both S89A and Y93A mutations showed palmitoyl-CoA hydrolase activity comparable with the wildtype protein (Fig. 6*B*). To further confirm these results, we assayed highly purified protein preparations of the wildtype and MBLAC2(D87A) enzymes generated in Sf9 cells and observed a similar inactivation of MBLAC2 acyl-CoA hydrolase activity for the MBLAC2(D87A) mutant (Fig. 6*C*), confirming that Asp87 is required for the catalytic activity of MBLAC2.

### MBLAC2 cross-links to an exogenous bifunctional lipid precursor in HEK-293 cells

**Figure 7:**
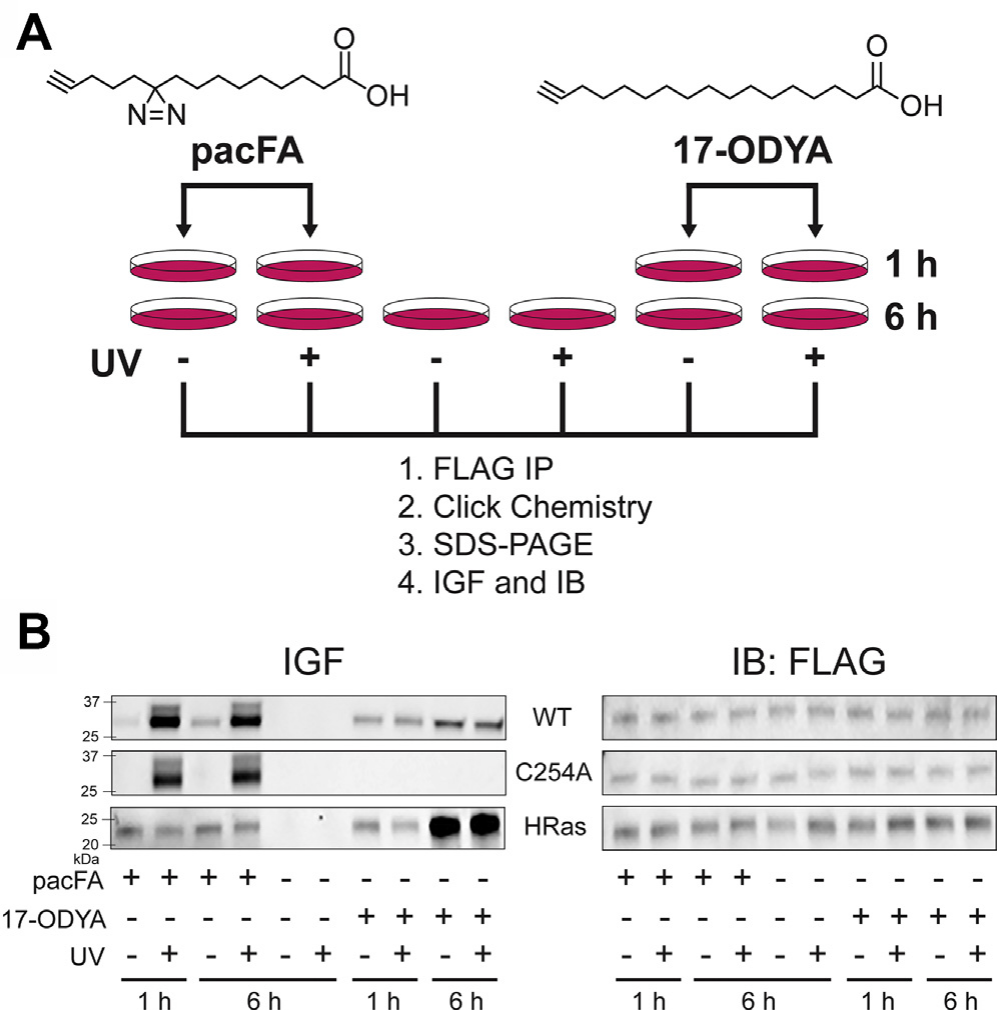
**MBLAC2 interacts with a photoactivatable and clickable fatty acid in cells independent of its S-palmitoylation.***A*, schematic diagram of the method used to detect lipid interaction of FLAG-MBLAC2 in HEK-293 cells. In this technique, transfected cells are incubated with photoactivatable and clickable fatty acid, pacFA, or a clickable palmitate analog, 17-ODYA, for 1 or 6 h. After cell lysis, FLAG-MBLAC2 is immunoprecipitated using FLAG resin and subjected to click chemistry. The protein is resolved by SDS-PAGE prior to label detection. *B*, lipid interaction of wildtype FLAG-MBLAC2 and MBLAC2(C254A) is measured by in-gel fluorescence (IGF) at 647 nm. Protein levels are compared by immunoblotting (IB) using a FLAG antibody. HRas, a dually palmitoylated protein, was used as control. The gel is representative of three independent experiments for the 1-h time points and two independent experiments for the 6-h time points.

We next examined if MBLAC2 can play a role in cellular lipid metabolism. To do this, we tested if MBLAC2 interacts with an exogenous lipid analog, pacFA (photoactivatable and clickable fatty acid). PacFA is bifunctional and contains two key features: a photoactivatable diazirine group that forms a covalent linkage to its protein-binding partners in proximity upon UV irradiation and a terminal alkyne group that allows for detection of protein–lipid complexes by click chemistry. When fed to cells, pacFA was demonstrated as a suitable precursor for the biosynthesis of bifunctional lipids such as phosphatidylcholine, phosphatidylenolamine, and phosphatidylinositol (31). Following transient transfection with N-terminally FLAG-tagged wildtype or MBLAC2(C254A), we fed HEK-293 cells with pacFA and then irradiated the cells with UV light to allow the formation of a chemical cross-link between the lipid and MBLAC2 contingent upon an interaction by proximity. Following immunoprecipitation with FLAG beads, the MBLAC2 protein from total cell lysates of HEK-293 cells was subjected to click chemistry and resolved by SDS-PAGE to detect pacFA-cross-linked MBLAC2 by in-gel fluorescence (Fig. 7*A*).

We detected pacFA-cross-linked wildtype MBLAC2 in pacFA-fed cells irradiated with UV for 1 and 6 h, with the levels of UV-irradiated labeling similar at both time points (Fig. 7*B*). A previous study showed that 1 h of metabolic labeling is sufficient for incorporation of the bulk of pacFA (95%) into various complex lipids such as diacylglycerol, triacylglycerol/cholesterol ester, and other phospholipids, whereas a longer labeling time allows for metabolic incorporation of pacFA as S-acylation (31). Because MBLAC2 is S-palmitoylated, we sought to distinguish whether the signal observed was a result of a lipid-protein cross-link or S-acylation. To do this, we included non-UV-irradiated pacFA-fed HEK-293 cells as a control. Not surprisingly, wildtype MBLAC2 showed little, but detectable, in-gel fluorescence in the absence of UV irradiation. This UV-independent labeling increased slightly with a longer pacFA incubation from 1 to 6 h, consistent with MBLAC2 being S-palmitoylated. A similar time-dependent increase in labeling was seen in cells labeled with 17-ODYA, a clickable palmitate analog. However, unlike the pacFA labeling, which increased dramatically upon UV irradiation at each time point, 17-ODYA labeling was not affected by UV irradiation. Notably, no labeling was observed when pacFA or 17-ODYA was omitted.

As expected, the S-palmitoylation-deficient MBLAC2(C254A) retained UV-irradiated pacFA labeling but lacked non-UV-irradiated pacFA labeling and any 17-ODYA labeling even with a 6-h incubation. In addition, HRas, not known to bind fatty acids or acyl CoAs, labeled with both pacFA and 17-ODYA labeling independent of UV irradiation, consistent with S-linked fatty acylation. Taken together, our data suggest that MBLAC2 interacts with the fatty acid independent of its S-palmitoylation.

## Discussion

**Figure 8:**
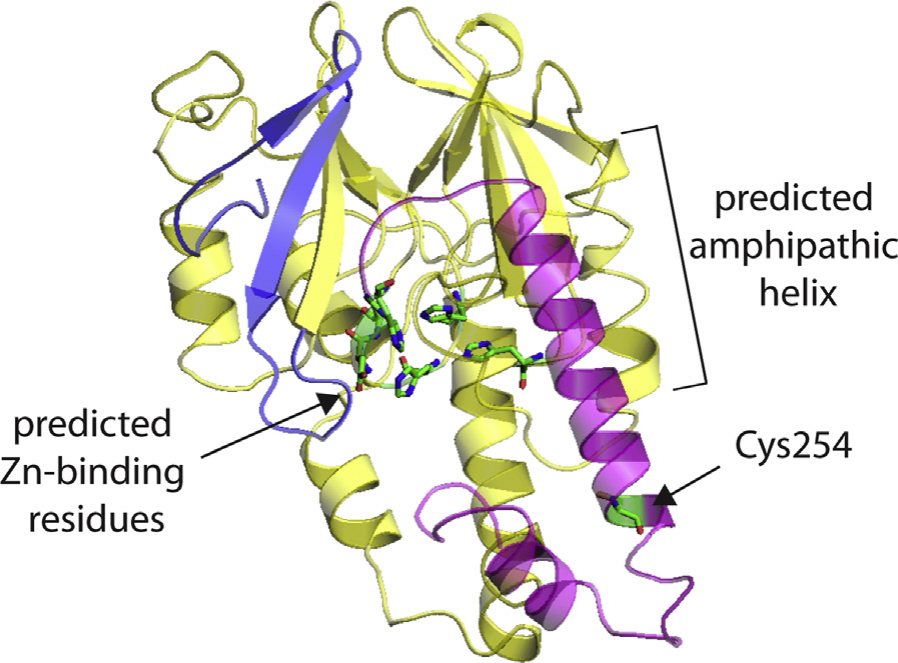
**Predicted three-dimensional fold of the human MBLAC2 protein.** Structure threading was performed in I-TASSER, a software that models unknown protein structures using templates available from the PDB library. Each candidate structure is given a C-score [-5 to 2], with 2 indicating the highest confidence. The highest C-score (0.2) obtained during the threading was for the crystal structure of β-lactamase domain protein from *Burkhoderia ambifaria* (PDB ID: 5I0P) (50, http://www.rcsb.org/structure/5I0P) and hence was used as a template in this figure. The resulting MBLAC2 structure was visualized using Pymol. The MBL-fold domain is represented by *yellow*, the N terminus by *blue*, and the C terminus by *magenta*. The predicted zinc-binding site residues in the active site are represented as sticks and colored by the element (C, *green*; N, *blue*; O, *red*). The Cys254 residue seen distant from the predicted Zn-binding active site is also colored by element. The predicted amphipathic helix within the MBL-fold domain is labeled (see discussion).

Our study provides the first biochemical characterization of MBLAC2 protein modification and function, enabling a better understanding of this member of the human MBL family. Evidence from palmitoylproteome screens and work herein suggests that MBLAC2 is unique among human MBL proteins in its status as a S-palmitoylated protein. We mapped the S-palmitoylation site in the protein to a cysteine residue approximately 20 amino acids from the carboxyl terminus, a region outside the predicted MBL fold (Fig. 8). We found that the S-palmitoylated cysteine has little or no effect on MBLAC2 membrane association or its subcellular distribution.

The question of what structural feature recruits MBLAC2 to the membrane remains unanswered. Aside from covalent lipid modifications, there are other mechanisms by which a soluble protein can associate with membranes. An example is an amphipathic α helix, a mechanism used by CTP:phosphocholine cytidylyltransferase (CCT) (32), prostaglandin endoperoxide H synthases (PGHs 1 and 2) (33, 34), and G protein-coupled receptor kinase 5 (GRK5) (35). Notably, the helix serves only as a reversible membrane anchor for CCT but facilitates the stable membrane association of PGH and GRK5. A subset of the regulators of G protein signaling (RGS) proteins have an amphipathic α helix within an N-terminal domain that mediates their membrane targeting (36). Of interest, RGS4 is palmitoylated at cysteine residues within this domain, but S-palmitoylation is a secondary event to membrane binding (37, 38, 39). We speculate that a predicted amphipathic helix in MBLAC2 (amino acids 205–218) may play a similar role in MBLAC2’s affinity for membranes. However, our ability to experimentally test this idea has been stymied by low expression levels and protein misfolding when mutations or deletions are introduced in this region of the protein, which is part of the MBL-fold (Fig. 8).

Our study revealed that MBLAC2 has robust acyl-CoA thioesterase activity *in vitro*. Acyl CoAs are widespread as intermediate substrates in diverse cellular metabolic pathways. These substrates are usually oxidized for energy production or incorporated into a variety of complex lipids (25). The cycle of esterification and de-esterification of fatty acids serves as a regulatory mechanism for controlled lipid metabolism. ACOTs are thought to account for most de-esterification of fatty acyl CoAs. MBLAC2 represents another enzyme that potentially plays a role in regulating cellular levels of acyl CoAs. MBLAC2 is distinct from the ACOTs with respect to mechanism. ACOTs are serine α/β hydrolases, whereas MBLAC2 is a zinc metalloenzyme.

We found that MBLAC2 displays a preference for hydrolyzing longer-chain acyl CoAs. This selectivity is remarkably similar to that of the zDHHC20 enzyme (30), suggesting that MBLAC2 may play a role in regulating the pool of acyl CoA available for the zDHHC20 enzyme. Such a mechanism has the potential to reduce zDHHC20 substrate S-palmitoylation and impact their S-palmitoylation-dependent functions. Relevant substrates for zDHHC20 include the epidermal growth factor receptor (EGFR) (40), the antiviral protein interferon-induced transmembrane protein 3 (IFITM3) (41), and the HIV-1 Tat protein (42).

Witze and co-workers reported that the C-terminal tail of EGFR is S-palmitoylated by zDHHC20. A reduction in zDHHC20 increases mitogen-activated protein kinase (MAPK) signaling by a mechanism that is independent of EGFR kinase activity (40). This study was followed by the demonstration that EGFR palmitoylation by zDHHC20 supports phosphatidylinositol 3-kinase (PI3K)-AKT signaling, leading to stable Myc production and cell proliferation (43). Loss of EGFR palmitoylation by silencing zDHHC20 leads to hyperactivation of KRAS-MAPK signaling and impedes PI3K-AKT signaling, causing Myc depletion and reduced cell proliferation. Of interest, reducing zDHHC20 levels impairs tumor formation in a KRAS-mutant mouse model. zDHHC20 reduction also arrests the growth of existing human, xenografted KRAS-mutant tumors in mice, heightening the interest in inhibiting zDHHC20-dependent EGFR S-palmitoylation as a potential clinical treatment strategy. Given that MBLAC2 and zDHHC20 interact with each other, codistribute in the same intracellular compartments, and display similar acyl CoA preference, we hypothesize that, in cells, MBLAC2 may serve to modulate the zDHHC20-dependent S-palmitoylation of its EGFR substrate and consequently the MAPK and PI3K signaling pathways. Similarly, MBLAC2 regulation of zDHHC20 activity could impact the antiviral response mediated by IFITM3, an activity that requires S-palmitoylation (44). Finally, a recent study reports that the HIV-1 Tat protein is S-palmitoylated by zDHHC20, a modification that is required for TAT accumulation at the plasma membrane where it affects PI(4,5)P_2_-dependent membrane traffic (42).

There is precedent for cellular levels of acyl CoAs influencing the activity of certain zDHHC proteins. Earlier work from our laboratory showed that zDHHC3 and zDHHC2 form S-palmitoylation-sensitive oligomers in cells using bioluminescence resonance energy transfer. Addition of palmitoyl CoA to cell membranes shifted the monomer–oligomer equilibrium in favor of the monomeric forms of these enzymes. This effect was not seen in the catalytically inactive zDHHS mutants (45). These results suggest that zDHHC protein activity may be regulated by the availability of palmitoyl CoA. This hypothesis is supported by results showing that palmitoyl CoA addition to unfractionated bovine brain membrane fractions significantly increased the total amount of S-palmitoylation *in vitro* (46).

Although we have assigned a biochemical function to MBLAC2 related to acyl-CoA metabolism, whether acyl CoAs represent the physiological substrate for MBLAC2 remains to be addressed. Our cross-linking experiments with a photoactivatable palmitate analog are consistent with MBLAC2 binding to fatty acids in cells. This association could represent an association with palmitoyl CoA, free fatty acid, or another lipid. A future goal is to determine whether exogenous expression or depletion of MBLAC2 results in changes in acyl-CoA levels in cells. Hydrolysis of acyl CoA yields free fatty acid and CoA. The possibility that MBLAC2 could facilitate the transfer of fatty acid to an acceptor molecule other than water should be considered, including cysteine thiols on proteins. However, preliminary experiments have not revealed facile transfer of the fatty acid to proteins (M.I.P.M. and M.E.L., unpublished results).

The roles of acyl-CoA hydrolase activity in regulating metabolism, particularly those enzymes localized in the cytoplasm are not well understood (25). ACOT1 expression is strongly induced by peroxisome proliferator-activated receptor α and hepatocyte nuclear factor 4α (47). It has been suggested that ACOT1 may regulate the pool of free long-chain fatty acids that act as ligands for nuclear receptors (25). Of interest, a recent report suggests that the other product of the acyl-CoA hydrolase reaction may function to regulate protein activity through protein modification (48). Proteins can undergo mixed disulfide bond formation with CoA, a process termed CoAlation. Metabolic enzymes are enriched in the CoAlated proteome, and modification of proteins with CoA is induced in mammalian cells and tissues by oxidizing agents and metabolic stress (48). Thus, acyl-CoA hydrolysis yields two moieties with the potential to affect protein function.

## Experimental Procedures

### Reagents

Anti-FLAG M2 affinity gel and 3X FLAG peptide were purchased from Sigma. A 1000x protease inhibitor cocktail of 5 mg/ml leupeptin (Sigma), 3 mg/ml aprotinin (Sigma), 1 M PMSF (MP Biomedicals) and 1 mM pepstatin A (Amresco) was mixed from individual components. Alexa Flour647 azide was purchased from Invitrogen. [^3^H]-palmitoyl CoA was synthesized as previously described (45). HEK-293 cells were cultured in Dulbecco’s modified Eagle medium (DMEM) (Gibco) media with 10% fetal bovine serum (Life Technologies). Cell were maintained at 37 ^°^C in a humidified incubator supplemented with 5% CO_2_. Polyethylenimine (PEI) (25k, linear) was purchased from Polysciences and was dissolved in water to make a 1000x stock (1 mg/ml). The following commercial antibodies were used: M2 FLAG (Sigma-Aldrich, F1804), MBLAC2 (Sigma-Aldrich, HPA037757), GFP (Invitrogen, A-11122), ER marker, calnexin (Cell Signaling, C5C9), nuclear marker, lamin (Sigma-Aldrich, SAB4200236), cytosolic marker, GAPDH (Sigma, G8795).

### Detection of S-palmitoylation in cells by click chemistry

HEK-293 cells transfected with MBLAC2 DNA using PEI reagent were cultured in DMEM at 37 °C. After 48 h, the media was replaced with DMEM containing 10% dialyzed FBS and 100 μM 17-ODYA (Cayman Chemical). The cells were returned to the incubator for 6 h. Cells were washed twice with phosphate-buffered saline (PBS) and then lysed with lysis buffer (50 mM Tris [pH 7.4], 100 mM NaCl, 10% glycerol, 1% DDM, protease inhibitors) for 30 min at 4 °C while rotating. Cleared lysates were diluted 10-fold with wash buffer (50 mM Tris [pH 7.4], 100 mM NaCl, 10% glycerol, 0.05% DDM) and immunoprecipitated with anti-FLAG resin for at least 4 h. The FLAG immunoprecipitates were washed three times with wash buffer and suspended in 90 μl of PBS. For click chemistry, 10 μl of freshly premixed click chemistry reagent (final concentrations of 10 μM Alexa Fluor 647-azide, 1 mM tris(2-carboxyethyl)phosphine [TCEP], 100 μM tris[(1-benzyl-1H-1,2,3-triazol-4-yl)methyl]amine [TBTA], and 1 mM CuSO_4_) was added. After 1 h at room temperature, the immunoprecipitates were washed twice with PBS and eluted with elution buffer (50 mM Tris [pH 7.4], 100 mM NaCl, 10% glycerol, 0.05% DDM, 100 μg/ml 3X FLAG peptide). FLAG elutions were treated with sample buffer for SDS-PAGE. Probe-labeled proteins were detected by in-gel fluorescence. Relative protein amounts were compared by immunoblots. To confirm the presence of thioester-linked S-palmitoylation in MBLAC2, FLAG elutions in sample buffer were incubated with either 1 M hydroxylamine (pH 8) or 1 M Tris (pH 8) for 1 h at 37 °C prior to SDS-PAGE and in-gel fluorescence detection.

### Coimmunoprecipitation

MBLAC2 and zDHHC constructs were cloned into the pCMV5-FLAG or pCGFP-EU2 vectors to include an N-terminal FLAG epitope or a C-terminal GFP tag, respectively. The day before transfection, HEK-293 cells were plated onto a 10-cm plate at 40% to 50% confluency. The cells were incubated overnight at 37 °C. The next day, the cells were transfected with 3 μg each of DNA constructs and 18 μg of PEI transfection reagent. After 48 h, the culture medium was aspirated and the cells were suspended and washed with 2 ml of ice-cold PBS. The cells were then disrupted in lysis buffers (50 mM Tris [pH 7.4], 200 mM NaCl, 1% DDM, 10% glycerol, 1 mM TCEP, protease inhibitors). The lysate was cleared by centrifugation at 100,000*g* for 40 min. The soluble fraction was incubated with pre-equilibrated FLAG resin for 4 h. The resin was washed with 10 x column volumes of wash buffer (100 mM Tris HCl [pH 7.4], 150 mM NaCl, 0.05% DDM, 0.5 mM TCEP). The FLAG-tagged proteins were then eluted with elution buffer (100 mM Tris HCl [pH 7.4], 150 mM NaCl, 100 μg/ml 3X FLAG peptide, 0.5 mM TCEP, 0.05% DDM). Relative protein amounts were compared by immunoblot.

### Two-step affinity purification of MBLAC2 enzymes

Recombinant baculovirus constructs expressing MBLAC2 were made using Bac-to-Bac baculovirus insect-cell expression system. MBLAC2 protein was purified similar to methods previously described for zDHHC enzymes (49). Sf9 cells were infected at 2.5 to 4.0 × 10^6^ cells/ml with MBLAC2 recombinant baculovirus at 27 °C for 48 h and then harvested by centrifugation at 500*g*. Cells were washed twice with ice-cold PBS and disrupted in lysis buffer (50 mM Tris [pH 7.4], 200 mM NaCl, 1% DDM, 10% glycerol, 1 mM TCEP, and protease inhibitors [0.5 μg/ml leupeptin, 3 μg/ml aprotinin, 0.3 μg/ml pepstatin A]). The lysate was cleared by centrifugation at 100,000*g* for 40 min. The soluble fraction was incubated with Ni-NTA agarose gel resin (Qiagen) and washed with 10 x column volumes of wash buffer (100 mM Tris [pH 7.4], 100 mM NaCl, 10% glycerol, 0.1 % DDM, 0.5 mM TCEP, 15 mM imidazole). The protein was eluted with elution buffer (50 mM Tris [pH 7.4], 100 mM NaCl, 10% glycerol, 0.05% DDM, 0.5 mM TCEP, 200–500 mM imidazole). Fractions containing the MBLAC2 protein were pooled and subsequently purified using Anti-FLAG M2 affinity gel and eluted with elution buffer (50 mM Tris [pH 7.4], 100 mM NaCl, 10% glycerol, 0.05% DDM, 100,100 μg/ml 3X FLAG peptide). The protein concentration was determined by plotting elution samples along a linear curve generated with known concentrations of bovine serum albumin (Sigma) stained with Coomassie gel stain and quantified using a VersaDocTM 5000 imaging system. Purified proteins were concentrated using Amicon Ultra filter units (Millipore-Sigma).

### Protein acyl transferase assay

Purified zDHHC20 enzyme (50 nM) was assayed in a 50-μl reaction with [^3^H]-palmitoyl CoA (1 μM) and MBLAC2 (1 μM) at 25 °C for 0 to 30 min. The reaction was stopped with the addition of 5X sample buffer containing 10 mM TCEP and resolved on a gel. Following staining of the gel with Coomassie blue, the substrate bands were excised, solubilized with 500 μl Soluene 500 (Perkin-Elmer), and heated at 37 °C overnight before being combined with 4.5 ml of Ultima Gold scintillation fluid (Perkin-Elmer) and quantified by scintillation spectroscopy.

### Subcellular fractionation

This procedure was adapted from a subcellular fractionation protocol used for MBLAC1, a human Group 1 MBL-fold enzyme (16). HEK-293 cells transfected with FLAG-MBLAC2 were washed in PBS, pelleted, suspended in a digitonin-containing buffer (50 mM Hepes [pH 7.4], 150 mM NaCl, 200 μg/ml digitonin), and incubated for 10 min at 25 °C while rotating. The cell suspension was subjected to centrifugation at 2000*g*, and the resultant supernatant was retained (cytosolic fraction). The pellet was suspended in an NP-40 buffer (50 mM Hepes [pH 7.4], 150 mM NaCl, 1% NP-40), and the cell lysate was left on ice in NP-40 buffer for 30 min, then centrifuged at 7000*g*. The resultant supernatant was retained and designated the membrane (organelle) fraction. The pellet was suspended in radio immuno precipitation assay buffer (50 mM Hepes [pH 7.4], 150 mM NaCl, 0.5% Na-deoxycholate, 0.1% SDS) and rotated for 1 h at 4 °C, then centrifuged for 10 min at 7000*g*. The supernatant was retained and designated the nuclear protein fraction. Each fraction was then subjected to immunoblot analysis.

### Immunofluorescence imaging

U87 or HEK-293 cells were seeded on 35-mm glass-bottom dishes (High Precision 1.5 coverslip, 14 mm glass diameter, MatTek) 24 h prior to transfection to ensure 30% to 50% confluency on the day of transfection. The cells were then transfected with 1 μg of MBLAC2 DNA with 3 μg of PEI transfection reagent. At 48 h, the cells were washed with PBS twice and fixed with 4% paraformaldehyde (Fisher) for 25 min. The cells were then permeabilized with 0.2% Triton X-100 (Sigma) in PBS for 15 min and blocked with 1% solution of bovine serum albumin BSA (Sigma) in PBS for 1 h. The plates were then incubated with the primary antibody followed by the secondary antibody for 1 h each with multiple washings in between. The following antibodies and markers were used: mouse monoclonal IgG1 anti-FLAG (1:500, Sigma), rabbit monoclonal IgG rabbit (1:50, Cell Signaling), NucBlue Live ReadyProbes Reagent (2 drops/ml, Thermo Fisher). The cells were then washed multiple times with PBS and kept at 4 °C. Imaging data were acquired through the Cornell University Biotechnology Resource Center on Zeiss LSM880 confocal/multiphoton microscope using a C-Apochromat 40x/1.2 W Korr FCS M27 objective. The Zen software was used for processing of all images.

### Acyl-CoA hydrolase assay

Palmitoyl CoA and other acyl CoAs were purchased from Sigma and stored at a stock concentration of 1 mM in 50 mM morpholinoethanesulfonic acid, pH 7.4 buffer containing 0.05% DDM. For the hydrolysis of palmitoyl CoA, a reaction hot mix (RHM) was prepared by mixing nonradioactive palmitoyl CoA with [^3^H]-palmitoyl CoA to a final concentration of 12.5 μM (specific activity 500 dpm/pmol). The reaction was initiated by adding 400 μl of the RHM to 100 μl of the enzyme solution (500 nM) at 30 °C. For a time-course experiment, the reaction was monitored for 30 min. For a single time point experiment, the reaction was terminated at 10 min with 1 ml of Dole’s reagent (2-propanol:heptane:1 M H_2_SO_4_, 25:5:1). The [^3^H]-palmitic acid product was isolated by extraction with 500 μl heptane followed by vigorous shaking for 1 h. The organic layer collected was quantified by scintillation spectrometry. For a competition assay, 1.25 μM [^3^H]-palmitoyl CoA was mixed with 11.25 μM of competing acyl CoA prior to adding the enzyme to form the RHM.

### Detection of lipid interaction in cells using a photoactivatable and clickable fatty acid

HEK-293 cells freshly plated in 10-cm dishes were transfected with FLAG-MBLAC2 DNA using the PEI transfection reagent and incubated with DMEM at 37 °C. After 24 h, the medium was replaced with DMEM containing 10% dialyzed FBS and 50 μM pacFA (Avanti Polar Lipids, Inc). A parallel experiment was performed using a clickable fatty acid, 17-ODYA. The cells were then incubated for an additional 1 or 6 h. For UV irradiation, cells were washed with 5 ml of PBS and irradiated using RAD-FREE long-wave UV lamp 365 nm (Schleicher and Schuell) for 10 min at room temperature. FLAG-MBLAC2 was then immunoprecipitated from the lysed cells and subjected to click chemistry as described above. Probe-labeled proteins were detected by in-gel fluorescence. Relative protein amounts were compared by immunoblots.

## Data availability

All data are contained in the article.

## Supporting information

This article contains supporting information (51, 52, 53).

## Conflict of interest

The authors declare that they have no conflicts of interest with the contents of this article.
