# Supplementary material for: Metallo-β-lactamase domain-containing protein 2 is S-palmitoylated and exhibits acyl-CoA hydrolase activity
Source: J Biol Chem. 2020 Dec 3;296:100106. doi: 10.1074/jbc.RA120.015701 (PMC7949124; doi:10.1074/jbc.RA120.015701)
Supplement: Fig. S1 [file mmc1.pdf]

Metallo-β-Lactamase Domain-Containing Protein 2 (MBLAC2) is S-palmitoylated and exhibits acyl-CoA hydrolase activity

Martin Ian P. Malgapo, Jenelle M. Safadi, Maurine E. Linder*

From the Department of Molecular Medicine, College of Veterinary Medicine, Cornell University, Ithaca NY, USA

Running title: *MBLAC2 is an acyl-CoA hydrolase*

*To whom correspondence should be addressed: Maurine E. Linder: Department of Molecular Medicine, College of Veterinary Medicine, Cornell University, Ithaca NY, USA; E-mail: mel237@cornell.edu

List of Supporting Information:

FIGURE S1. MBLAC2 showed little or no activity as a hydrolase of various small-molecule and thioacylated-protein substrates.

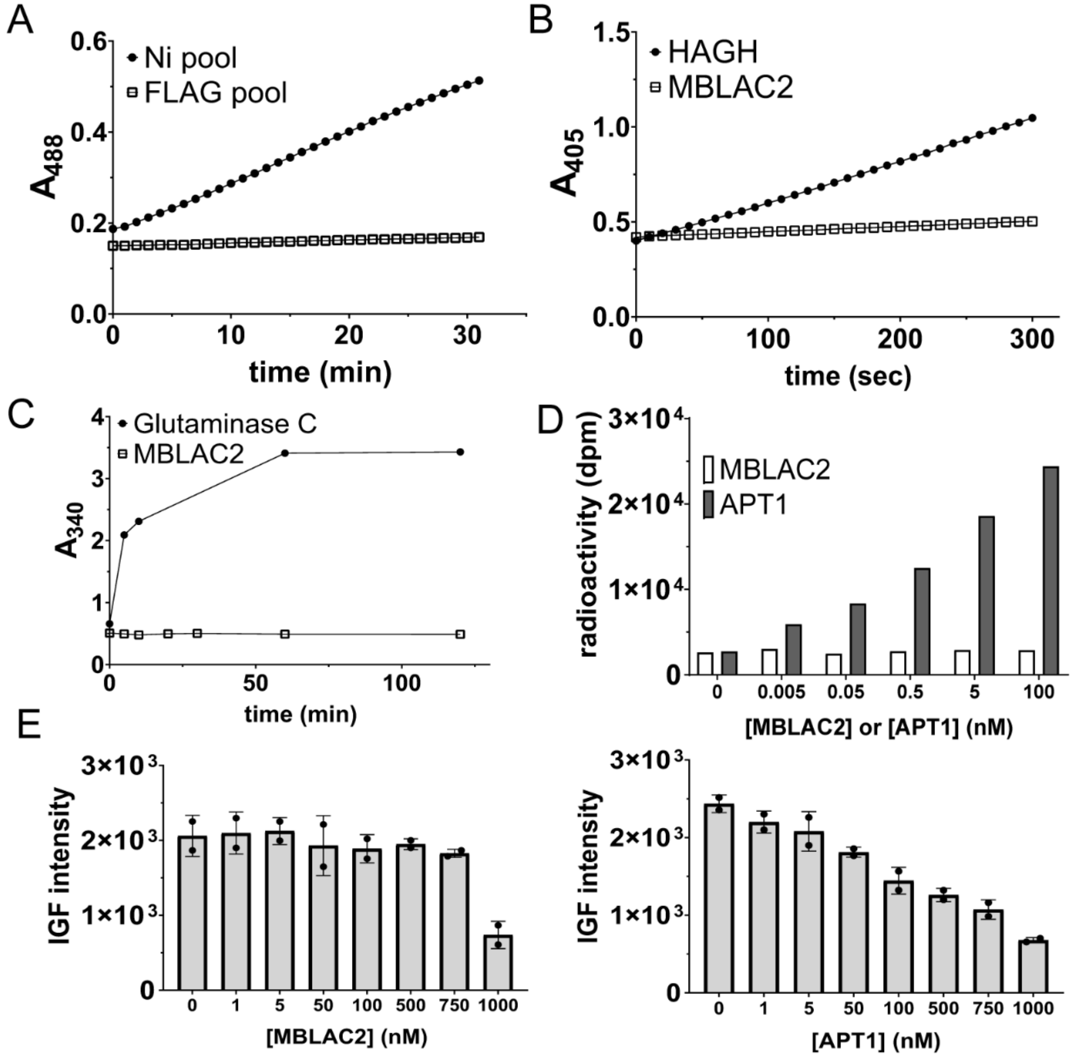

FIGURE S1. MBLAC2 showed little to no activity as a hydrolase to various small molecule and thioacylated protein substrates.

A. Nitrocefin hydrolysis assay [21]. MBLAC2 (1 µM) purified by either one step- (Ni pool) or two step- (FLAG pool) affinity chromatography was used to hydrolyze nitrocefin (1 mM) at 30 °C for 30 min. The hydrolysis reaction was monitored by measuring the absorbance of red-colored product at 488 nm. The nitrocefin amidase activity seen in the MBLAC2 Ni pool was undetectable in MBLAC2 FLAG pool. The data shown is representative of two independent experiments.
B. S-lactoyl-glutathione hydrolysis assay. MBLAC2 FLAG pool (50 nM) or commercial recombinant HAGH (50 nM, positive control) were used to hydrolyze S-lactoyl-glutathione (1 mM) at 30 °C for 5 min using a colorimetric Glyoxalase II Assay Kit (BioVision). The hydrolysis reaction was monitored by measuring the absorbance of the lactate product at 488 nm. The data shown are representative of two independent experiments.
C. Glutaminase assay [52]. MBLAC2 FLAG pool (50 nM) or Glutaminase C (50 nM, positive control) was used to hydrolyze glutamine (20 nM) at room temperature. The reaction was monitored by measuring the absorbance at 340 nm of the hydrolyzed carboxylic acid product. The data shown are from one experiment.
D. Protein Thioesterase Assay on SH4-GFP [53]. Src-homology domain 4 (SH4) is an N-myristoylated model substrate containing three cysteine residues [22]. Purified [^{3}H]-palmitate-labeled SH4-GFP (1 µM) was incubated with various concentrations of MBLAC2 or APT1 at 25 °C for 10 min to initiate hydrolysis. The [^{3}H]-palmitic acid released from SH4-GFP was then extracted and the radioactivity was quantified by scintillation spectroscopy as described under

Experimental Procedures. The data shown are representative of two independent experiments. Data are displayed as mean +/- SEM, n=2.

E. Protein Thioesterase Assay on HRas [54]. Purified 17-ODYA-labeled HRas (1 μM) was incubated with various concentrations of MBLAC2 FLAG pool (left panel) or acylprotein thioesterase 1 (APT1) (right panel, positive control) at 25 °C for 10 min to initiate hydrolysis. After a click chemistry reaction and gel electrophoresis, the 17-ODYA labeling that remained in HRas was measured by in-gel fluorescence (IGF). The data shown are representative of two independent experiments. Data are displayed as mean +/- SEM, n=2.
